# Highly Flexibility, Powder Self-Healing, and Recyclable Natural Polymer Hydrogels

**DOI:** 10.3390/gels8020089

**Published:** 2022-01-31

**Authors:** Haiyue Miao, Weiju Hao, Hongtao Liu, Yiyang Liu, Xiaobin Fu, Hailong Huang, Min Ge, Yuan Qian

**Affiliations:** 1 School of Materials and Chemistry, University of Shanghai for Science and Technology, No. 516 Jungong Road, Shanghai 200093, China; miaohaiyue@sinap.ac.cn (H.M.); wjhao@usst.edu.cn (W.H.); 2 Key Laboratory of Interfacial Physics and Technology, Department of Molten Salt Chemistry and Engineering, Shanghai Institute of Applied Physics, Chinese Academy of Sciences, Shanghai 201800, China; liuhongtao@sinap.ac.cn (H.L.); liuyiyang@sinap.ac.cn (Y.L.); gemin@sinap.ac.cn (M.G.)

**Keywords:** hydrogels, flexibility, self-healing, recyclability

## Abstract

Based on the good self-healing ability to repair mechanical damage, self-healing hydrogels have aroused great interest and been extensively applied as functional materials. However, when partial failure of hydrogels caused by breaking or dryness occurs, leading to recycling problems, self-healing hydrogels cannot solve the mentioned defects and have to be abandoned. In this work, a novel recyclable and self-healing natural polymer hydrogel (Chitosan/polymethylacrylic acid-: CMA) was prepared. The CMA hydrogel not only exhibited controlled mechanical properties from 26 kPa to 125 kPa with tensile strain from 1357% to 3012%, but also had good water retaining property, stability and fast self-healing properties in 1 min. More importantly, the CMA hydrogel displayed attractive powder self-healing performance. After drying–powdering treatment, the mentioned abandoned hydrogels could easily rebuild their frame structure to recover their original state and performance in 1 min only by adding a small amount of water, which could significantly prolong their service life. These advantages guarantee the hydrogel can effectively defend against reversible mechanical damage, water loss and partial hydrogel failure, suggesting great potential applications as a recyclable functional hydrogel for biomaterials and electronic materials.

## 1. Introduction

Hydrogels are water-swollen polymeric materials displaying good hydrophilic three-dimensional (3D) networks by chemical or physical crossing links [1,2,3]. Owing to their peculiar structure, hydrogels exhibits characteristic properties [4,5,6], (e.g., high softness, hydrophilic nature, insolubility, swelling behavior and sensitivity to physiological environment). Over recent decades, hydrogel-based functional materials have aroused great interest and been extensively applied in adsorption materials, as drug carriers, in tissue engineering and wearable flexible electronic devices [7,8,9,10,11,12,13,14]. However, although hydrogels exhibit obvious advantages in performance, they are abandoned after suffering irreversible damage (e.g., mechanical breaks, dryness or when part of the hydrogel is missing). Due to the lack of effective recycling strategies, the abandoned materials cause serious waste and environment pollution [15,16]. Thus, a novel recyclable hydrogel with good performance should be developed.

In recent years, various functional hydrogels have been designed to try to solve these problems. Self-healing hydrogel is considered to be an ideal candidate to address mechanical damage [17,18,19,20]. When impacted by reversible and dynamic interactions, self-healing hydrogel can effectively rebuild the hydrogel’s frame structure and recover its performance. Jiang et al. [21] prepared a copolymer hydrogel (poly(MAA-co-BA-co-OEGMA): methacrylic acid/oligo(ethylene glycol) methacrylate/4-Hydroxybenzaldehyde/ethylenediamine) with good self-healing abilities, based on dynamic hydrogen bonds and imine bonds. The cut hydrogel could rapidly merge into one piece in 2 min and recover 87% of the mechanical strength of its original state at 25 °C after 40 min. However, when hydrogels lose water, they cannot maintain good self-healing ability and have to be abandoned. To solve the dryness problem, Pan et al. [22] used vinylimidazole and hydroxypropyl acrylate to prepare a good self-healing hydrogel with excellent swelling–shrinking properties. The hydrogel had good self-healing ability, while exhibiting good wetness. Based on an elastic frame structure, the hydrogel could recover from a dry state to its original state with addition of water. After 5 swelling–shrinking cycles, it still could maintain stable mechanical performance, but once parts of the hydrogel were missing, the remaining hydrogel could not recover to its original state and maintain stable performance. In a recent study by our groups, a recyclable hydrogel (sodium alginate/polymethyl-acrylic acid) was developed with good self-healing ability and powder self-healing performance [23]. Under reversible and dynamic interactions, the hydrogel was capable of preventing mechanical damage and the dryness problem, as well as having good recyclability through drying–powdering treatment. Although the powder self-healing SAMA hydrogel exhibited good recyclability, its mechanical properties and self-healing response time are not enough to meet the requirements of applications. Accordingly, a new hydrogel material exhibiting good mechanical properties and fast powder self-healing properties should ideally be developed to recycle hydrogel materials, although this remains highly challenging.

In this work, a novel recyclable natural polymer hydrogel (CMA: chitosan/polymethyl-acrylic acid) was designed and prepared. The CMA hydrogel exhibited good mechanical properties under 26 kPa to 125 kPa with tensile strain from 1357% to 3012%, and it could be controlled by regulating the content of hydrogel. Besides, it displayed good self-healing ability, and could effectively heal in 1 min and recover 92.9% mechanical strength of its original state after 5 min at room temperature. More importantly, the CMA hydrogel possessed excellent powder self-healing performance. Failed hydrogel could be easily recycled based on drying–powdering treatment, which could effectively solve the problems of dryness, partially missing hydrogel and recycling problems to prolong the service life in 1 min. Benefiting from the mentioned advantages, this powder self-healing hydrogel will have great potential application as a recyclable functional hydrogel in biomaterials and electronic materials.

## 2. Results and Discussion

### 2.1. Characterization of CMA Hydrogels

**Figure 1:**
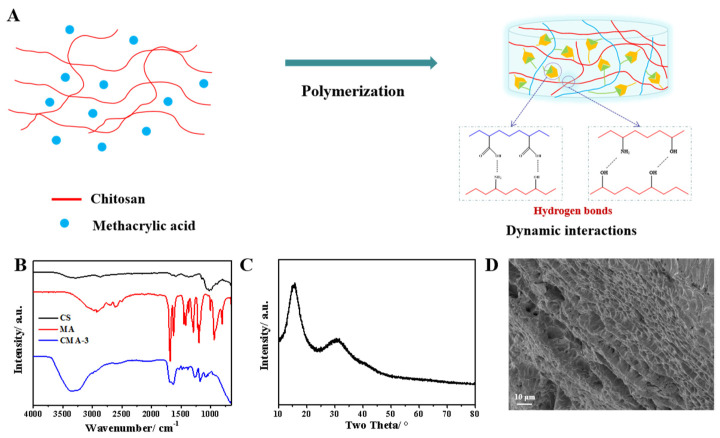
(**A**) Preparation of CMA hydrogels. (**B**) FTIR spectra of CS, MA and CMA-3 hydrogels. (**C**) XRD pattern of CMA-3 hydrogel. (**D**) SEM image of CMA-3 hydrogel.

Figure 1A illustrates the preparation of the natural polymer hydrogel. Based on the natural frame structure of Chitosan (CS) and functional monomer-methacrylic acid (MA), CMA hydrogels were synthesized through free radical polymerization by reversible hydrogen bonds with 3D network structures. The chemical structure displayed by the CMA hydrogels was analyzed by Fourier-transform infrared spectroscopy (FTIR) and X-ray diffraction (XRD). According to Figure 1B, the broad peak at 3247 cm^−1^ corresponded to the –OH and –NH_2_ stretching vibration. The peaks at 1642 cm^−1^ and 1588 cm^−1^ belonged to the –C=O and -NH bending vibration [24,25]. Moreover, the characteristic peaks of methacrylic acid (MA) at 3018 cm^−1^, 1686 cm^−1^ and 1622 cm^−1^, respectively, belonged to the stretching vibrations of –C=C, –COOH and –C=C. In the spectra of the CMA hydrogels, all the characteristic peaks of CS and MA could be observed. Besides, the significant shifting peaks from 3247 cm^−1^ to 3360 cm^−1^ and from 1588 cm^−1^ to 1637 cm^−1^ indicated the strong dynamic hydrogen bonds. The mentioned results demonstrate the successful preparation of the CMA hydrogels. Figure 1C shows the XRD pattern of the CMA hydrogels. The two broad characteristic peaks at 2θ = 15° and 2θ = 30° belong to the amorphous structure of the hydrogels. As shown in Figure 1D, the micro-morphology of the CMA hydrogels exhibited a continuous and regular porous structure, which would be conducive to maintaining good flexibility and water retaining ability of the hydrogels.

### 2.2. Mechanical and Water Retaining Properties of CMA Hydrogels

**Figure 2:**
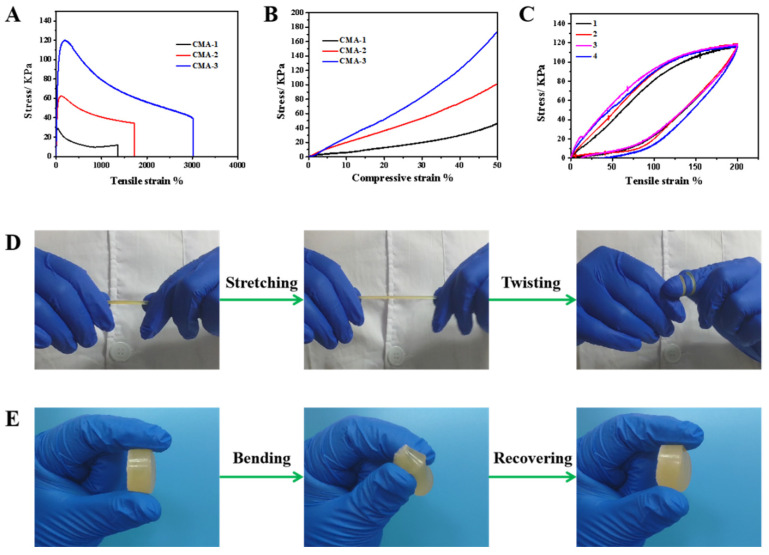
Mechanical properties of CMA hydrogels: (**A**) Tensile strain curve at 10 mm min^−1^. (**B**) Compressive strain curve at 5 mm min^−1^. (**C**) Stretching–releasing cycles. Photographs of CMA-3 hydrogel exhibiting excellent stretching and bending flexibility (**D**,**E**).

Mechanical properties are a vital performance parameter against which to assess hydrogel materials. The mechanical strength of CMA hydrogels was analyzed through performing stress strain experiments. As shown in Figure 2A, all the CMA hydrogels exhibited good stretching ability. With the increase of MA content from 1.0 g to 3.0 g (Table S1), the stretching stress increased from 26 kPa to 125 kPa, with tensile strain increasing from 1357% to 3012%, and Young’s modulus from 20.3 kPa to 42.9 kPa. Besides, the compressing ability of the CMA hydrogels showed excellent performance. With the increase of MA content from 1.0 g to 3.0 g, the compressing stress increased from 32 kPa to 176 kPa under 50% compressing strain (Figure 2B). The CMA hydrogels demonstrated controlled mechanical properties to meet different application needs (Table S2, Figure S1). With different contents of MA, the CMA-3 hydrogel showed optimal mechanical properties with 3012% stretching strain under 125 kPa and 50% compressing strain under 176 kPa. Compared with previously reported self-healing hydrogels, especially in hydrogel biomaterials and hydrogel electrolytes, the CMA-3 hydrogel exhibited much better mechanical properties (Table S3) [26,27,28,29,30,31,32,33]. Besides, it showed good flexibility. During 4 stretching–recovering cycles, although the stress–strain curves exhibited a small hysteresis loop, the hydrogel could quickly recover to its original shape when the stress released, suggesting excellent self-recovery ability (Figure 2C).

This could be attributed to the continuous porous structure and strong reversible hydrogen interactions, which could effectively counteract the effect external force to enhance its mechanical properties [34,35,36]. To clearly show the gel’s flexibility, the prepared CMA-3 hydrogel stick and disk were used as demonstrations. As shown in Figure 2D,E, in the continuous stretching and bending processes, the CMA-3 hydrogel exhibited good flexibility without breaking. Besides, it showed excellent compressing ability. Even under a human weight stress (Figure S2), the hydrogel could maintain stability without breaking and immediately recover to its original state when the load was removed. Due to its excellent mechanical properties, the CMA-3 hydrogel was used for the following tests.

Its water-retaining property is another important factor for hydrogel materials. To assess its stability, the water loss process of CMA-3 hydrogel was recorded for 48 h at room temperature of 20 °C under 70% RH. As shown in Figure S3, the CMA-3 hydrogel could maintain more than 85% water after 48 h, which could meet most applications’ needs. This could be attributed to the many hydrophilic groups in the hydrogel, which could effectively block the water movement and protect the water from evaporation.

### 2.3. Self-Healing Property of CMA-*3* Hydrogel

**Figure 3:**
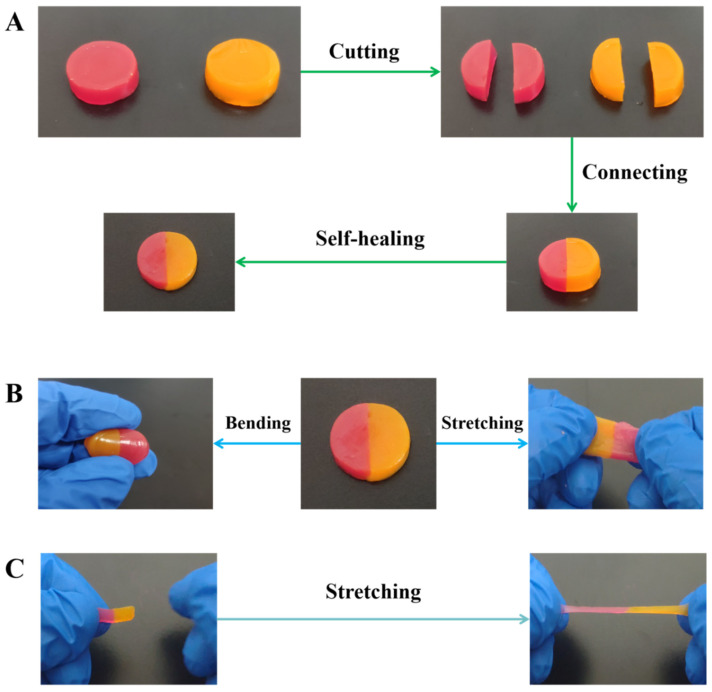
(**A**) Photographs of the self-healing abilities of CMA-3 hydrogel (hydrogel disks colored by rhodamine 6G and rhodamine B). Photographs of CMA-3 hydrogel exhibiting excellent stretching and bending flexibility (**B**,**C**).

Under reversible hydrogen bonds, the CMA-3 hydrogels exhibited good self-healing ability. To directly demonstrate this self-healing performance, two CMA-3 hydrogel disks were prepared and colored with rhodamine B and rhodamine 6G, respectively. First, the colored hydrogel disks were cut into two pieces. Subsequently, the red half and the orange half were placed in contact along the cutting line and maintained stability at room temperature without any external stimulus or healing agent. After 1 min, it was found that the two half hydrogels successfully self-healed into a single one (Figure 3A).

Additionally, the healed hydrogel exhibited good mechanical strength to withstand continuous twisting and stretching without cracking, demonstrating good ability to recover its mechanical properties (Figure 3B,C). In order to quantitatively assess the self-healing property of the hydrogel, the mechanical strength changes over time of the CMA-3 hydrogel were tested during the self-healing process. As shown in Figure S4A,B, with the increase of self-healing time, the mechanical property gradually recovered. After 5 min, the CMA-3 hydrogel could recover nearly 92.9% strain and 82.7% stress of its original state, respectively. Compared with previously reported self-healing hydrogels, the CMA-3 hydrogels showed much better self-healing properties (Table S3), which could be attributed to the reversible hydrogen bonds. When the hydrogels were cut, considerable reversible interactions were broken, and numerous functional groups separated and exposed on the cutting surface. While the cut hydrogel pieces were put together, the exposed functional groups would immediately interact with each other via hydrogen bonds, and efficiently rebuild the frame structure to realize the hydrogels’ self-healing.

To further demonstrate the self-healing property of the CMA-3 hydrogel, the colored hydrogels were cut into small particles as shown in Figure S5. Subsequently, these particles were put into a heart-shaped mold to record the self-healing process. Within 10 min, a complete heart-shaped hydrogel was obtained with good flexibility. These results indicated that the CMA-3 hydrogels possessed excellent self-healing property, which could effectively defend irreversible mechanical damage to prolong their service life.

### 2.4. Powder Self-Healing Property of CMA-*3* Hydrogel

**Figure 4:**
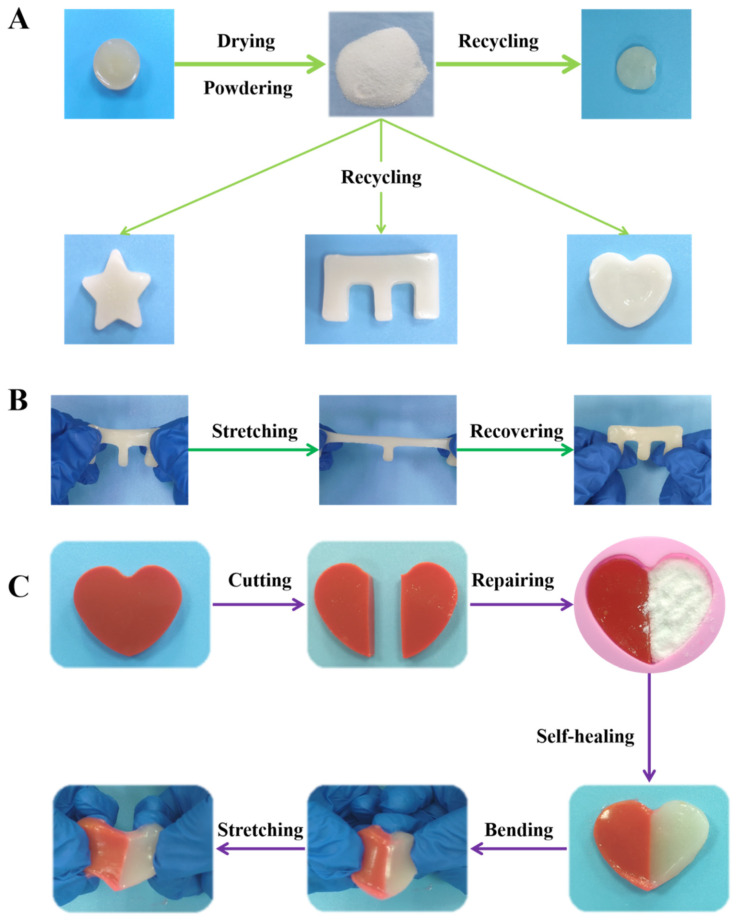
(**A**) Photographs of powder self-healing abilities of CMA-3 hydrogel. (**B**) Photographs of CMA-3 hydrogel exhibiting excellent stretching flexibility. (**C**) Photographs of CMA-3 hydrogel repairing partially missing hydrogel.

Dryness is a serious problem of hydrogels. Once water is lost, the hydrogel will quickly dry and shrink, resulting in performance failure, which significantly narrows the application of hydrogels. Fortunately, under strong reversible interactions, the CMA-3 hydrogel exhibited an excellent powder self-healing property, which could effectively solve the dryness problem of hydrogels. As shown in Figure 4A, the failed CMA-3 hydrogel was dried and powdered. Then, the powder was added into the round shape mold with a little water at room temperature. After 1 min, the powder quickly formed a piece of hydrogel with the mold shape (SI-V1). This is attributed to the benefit of the strong dynamic hydrogen bonds in the hydrogels. The added water served as a necessary medium to help the dry hydrogel powder rebuild the frame structure through strong dynamic interactions. 

To further verify its good powder self-healing property, the recycled CMA-3 hydrogel was dried and powdered again. The powder was put into different shaped molds to recycle the hydrogel. As shown in Figure 4A, the recycled powder could regenerate the hydrogels with various mold shapes, which indicated good recyclability. It was noteworthy that the recycled hydrogel also recovered its mechanical strength (Figure 4B). Under the continuous stretching–releasing process, the recycled hydrogel could maintain good flexibility. Based on this excellent powder self-healing performance, the CMA-3 hydrogel could easily address the dryness defect of hydrogels, suggesting good recyclable ability.

Besides, due to its powder self-healing ability, the hydrogel not only could address the dryness problem, but serve as an agent to repair hydrogels with parts missing. By adding a little prepared hydrogel powder and water, it could quickly repair the defects and reform a complete hydrogel in 5 min. Meanwhile, the repaired hydrogel kept good flexibility. As shown in Figure 4C, although a fracture could be observed at the contact position, the self-healed hydrogel exhibited good mechanical properties to withstand the tensile force without splitting. Based on the excellent advantages of good flexibility, self-healing ability and powder self-healing performance, the CMA hydrogel could effectively overcome the defects of mechanical damage, dryness and missing parts, which lead to the performance failure of hydrogel materials and their abandonment. More importantly, the attractive recyclability could guarantee the sustainability of hydrogel materials to prolong their service life.

## 3. Conclusions

In this work, we designed and prepared a novel highly flexible, self-healing and recyclable natural polymer hydrogel. The CMA hydrogel exhibited good flexibility and controlled mechanical properties with the content of MA (tensile strain from 1357% to 3012% under 26 kPa to 125 kPa, and 50% compressive strain under 32 kPa to 176 kPa). Besides, the CMA-3 hydrogel demonstrated remarkable self-healing ability, which could effectively repair the mechanically damaged hydrogel in 1 min and recover 92.9% mechanical strength of its original state in 5 min. More importantly, due to its reversible and dynamic interactions, the CMA-3 hydrogel showed excellent powder self-healing performance. The failure of hydrogel caused by dryness or missing parts could be easily repaired and a complete hydrogel rebuilt with the required shape in 1 min after drying–powdering and addition of water, and its mechanical strength recovered, indicating excellent recyclability. Based on these advantages, the CMA hydrogel should be potentially useful in diverse areas such as tissue engineering, stretchable electronics and wearable or implantable devices.

## 4. Materials and Methods

### 4.1. Materials

Chitosan, Methacrylic acid, rhodamine 6G, brilliant green, rhodamine B and ammonium persulfate (APS) were purchased from Sinopharm Chemical Reagent Co., Ltd., Shanghai, China.

### 4.2. Preparation of CMA Hydrogels

A total of 4.0 g of CS aqueous solution (4%, wt) and 3.0 g of MA were mixed together with stirring at room temperature for 6 h. Subsequently, 0.05 g of APS was added into the CS-MA solution while keeping it stirring for 1 h. After that, the mixture was added into a prepared mold and heated at 58 °C. After 6 h, the CMA hydrogels were formed. In order to regulate the mechanical properties, CMA hydrogels with different contents of MA were prepared, as shown in Table S1.

### 4.3. Characterization

The Fourier transform infrared spectroscopy spectrum (FTIR) of CMA hydrogels were measured on PerkinElmer Frontier (Waltham, Massachusetts, USA) at room temperature. The X-ray diffraction pattern (XRD) of CMA hydrogels were analyzed via X-ray diffractometer (Holland Panalytical PRO PW 3040/60, V = 35 kV, I = 25 mA, λ = 1.5418 Å, Eindhoven, The Netherlands), in the 2θ range of 10–80° at a scanning rate of 10° min^−1^. The micro morphology of CMA-3 hydrogels was characterized by scanning electron microscope (SEM, Hitachi-4800, Tokyo, Japan). The mechanical properties of CMA hydrogels were displayed on the tensile stress machine (Instron 5967, Boston, MA, USA). To perform stretching strain experiments, the tensile strain rate reached 10.0 mm·min^−1^, and the compressed strain rate was at 5.0 mm·min^−1^ under a strain range of 0–50% for the compressing strain experiments. The water retaining property of CMA hydrogels was measured at room temperature (20 °C) under 75% relative humidity.

In order to directly demonstrate the self-healing, the CMA-3 hydrogels were colored by organic dye. Two cylindrical CMA-3 hydrogels were colored by rhodamine 6G and brilliant green and were cut in half. Then, two different colored semicircular samples were put together and kept stable for 1 min at room temperature. The photographs were taken to record the self-healing process of hydrogel.

Tensile stress strain curves of the CMA hydrogel were recorded at a deformation rate of 10 mm/min at 25 °C on a tensile machine (Instron 5967, Boston, MA, USA), respectively. The original and the self-healed hydrogels were examined. For the self-healed hydrogels, the load was applied at the joint surface of two semicircular hydrogels.

## Data Availability

The data presented in this study are available in the article.

## Supplementary Materials

The following supporting information can be downloaded at: https://www.mdpi.com/article/10.3390/gels8020089/s1, Table S1: Different contents of CMA hydrogels; Table S2: Mechanical properties of CMA hydrogels; Table S3: Comparisons of self-healing ability of CMA-3 hydrogel with previous reported self-healing hydrogel in the literature; Figure S1: Comparisons of Young modulus of CMA-3 hydrogel with previous reported self-healing hydrogel in the literature; Figure S2: Compressing-recovering process of CMA-3 hydrogel; Figure S3: Water retaining ability of CMA-3 hydrogel; Figure S4: Tensile stress–strain curves of CMA-3 hydrogel during the self-healing process at different healing time (**A,B**); Figure S5: Photographs of self-healing ability of CMA-3 hydrogel.
