# Supplementary material for: Highly Flexibility, Powder Self-Healing, and Recyclable Natural Polymer Hydrogels
Source: Gels. 2022 Jan 31;8(2):89. doi: 10.3390/gels8020089 (PMC8871090; doi:10.3390/gels8020089)
Supplement: Supplementary file 1 [file gels-08-00089-s001.zip › gels-1518004-supplementary/gels-1518004-supplementary-updated.pdf]

gels

Supplementary

Highly Flexibility, Powder Self-Healing, and Recyclable Natural Polymer Hydrogels

Haiyue Miao [1,2,†], Weiju Hao [1,†], Hongtao Liu [2], Yiyang Liu [2], Xiaobin Fu [2,*], Hailong Huang [2,*], Min Ge [2] and Yuan Qian [2,*]

[1] School of Materials and Chemistry, University of Shanghai for Science and Technology, No. 516 Jungong Road, Shanghai 200093, China; miaohaiyue@sinap.ac.cn (H.M.); wjhao@usst.edu.cn (W.H.)

[2] Key Laboratory of Interfacial Physics and Technology and Department of Molten Salt Chemistry and Engineering, Shanghai Institute of Applied Physics, Chinese Academy of Sciences, Shanghai 201800, China; liuhongtao@sinap.ac.cn (H.L.); liuyiyang@sinap.ac.cn (Y.L.); gemin@sinap.ac.cn (M.G.)

* Correspondence: fuxiaobin@sinap.ac.cn (X.F.); huanghailong@sinap.ac.cn (H.H.); qianyuan@sinap.ac.cn (Y.Q.)

† These two authors contributed equally to this work.

Citation: Miao, H.; Hao, W.; Liu, H.; Liu, Y.; Fu, X.; Huang, H.; Ge, M.; Qian, Y. Highly Flexibility, Powder Self-Healing, and Recyclable Natural Polymer Hydrogels. *Gels* **2022**, *8*, 89. https://doi.org/10.3390/gels8020089

Academic Editor: David Mills

Received: 4 December 2021
Accepted: 12 January 2022
Published: 31 January 2022

Publisher's Note: MDPI stays neutral with regard to jurisdictional claims in published maps and institutional affiliations.

Copyright: © 2022 by the authors. Licensee MDPI, Basel, Switzerland. This article is an open access article distributed under the terms and conditions of the Creative Commons Attribution (CC BY) license (https://creativecommons.org/licenses/by/4.0/).

Table S1. Different contents of CMA hydrogels.

Sample	CS (g)	MA (g)	APS (g)	Heating Time (h)
CMA-1	4.0	1.0	0.05	6.0
CMA-2	4.0	2.0	0.05	6.0
CMA-3	4.0	3.0	0.05	6.0

Table S2. Mechanical properties of CMA hydrogels.

Sample	Tensile strain	Stress	Young modulus	Toughness
CMA-1	1357%	26 k·Pa	20.3 k·Pa	0.18 MJ/m^3
CMA-2	1718%	62 k·Pa	78.5 k·Pa	0.76 MJ/m^3
CMA-3	3012%	125 k·Pa	129.4 k·Pa	2.14 MJ/m^3

Table S3. Comparisons of self-healing ability of CMA-3 hydrogel with previous reported self-healing hydrogel in the literature.

Sample	Tensile strain	Self-healing time/rate	Dryness	Partial of hydrogel missing	Recyclability	Ref.
PPBN	991%	6.0 h/90%	x	x	x	[1]
IPN	1400%	24.0 h/80%	√	x	x	[2]
PVA/PEGDA/Agar/H_2SO_4	431%	24.0 h/52.4%	x	x	x	[3]
BH-5.5-1.5-0.4	645%	24.0 h/90%	x	x	x	[4]
JGNs/PAA	508.5%	1.0 h/93.4%	x	x	x	[5]
CNCs@P4VP	921.6%	6.0 h/85.9%	x	x	x	[6]
PAA-CMC-Al^{3+}	2066%	1.0 h/96.3%	x	x	x	[7]
TOCNF-GN/PAA	850%	12.0 h/96.7%	x	x	x	[8]
CMA	3020%	5 min/92.9%	√	√	√	This work

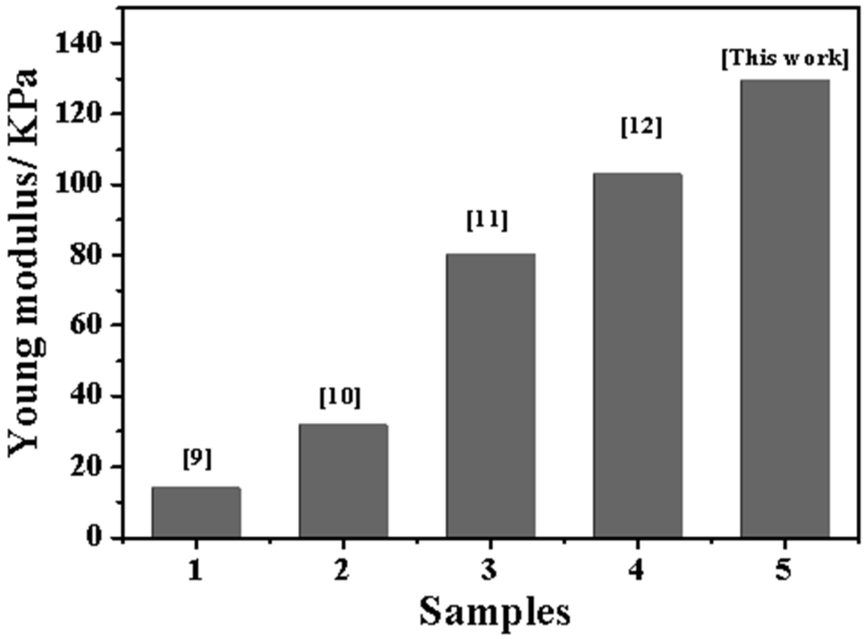

Figure S1. Comparisons of Young modulus of CMA-3 hydrogel with previous reported self-healing hydrogel in the literature.

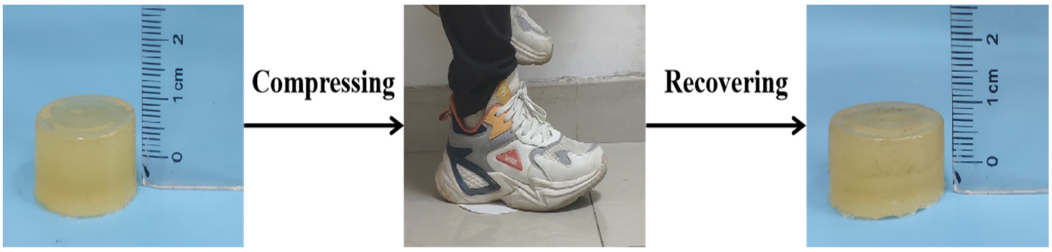

Figure S2. Compressing-recovering process of CMA-3 hydrogel.

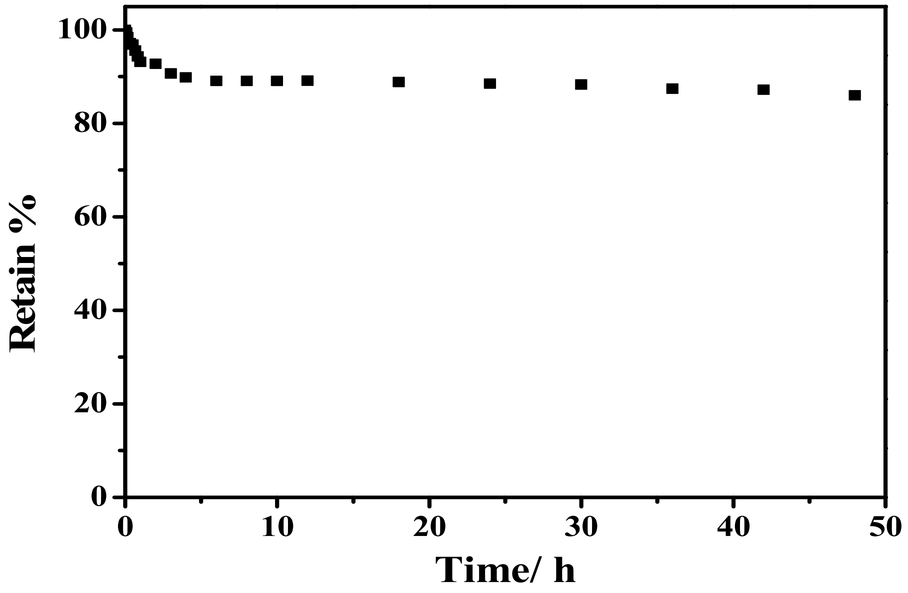

Figure S3. Water retaining ability of CMA-3 hydrogel.

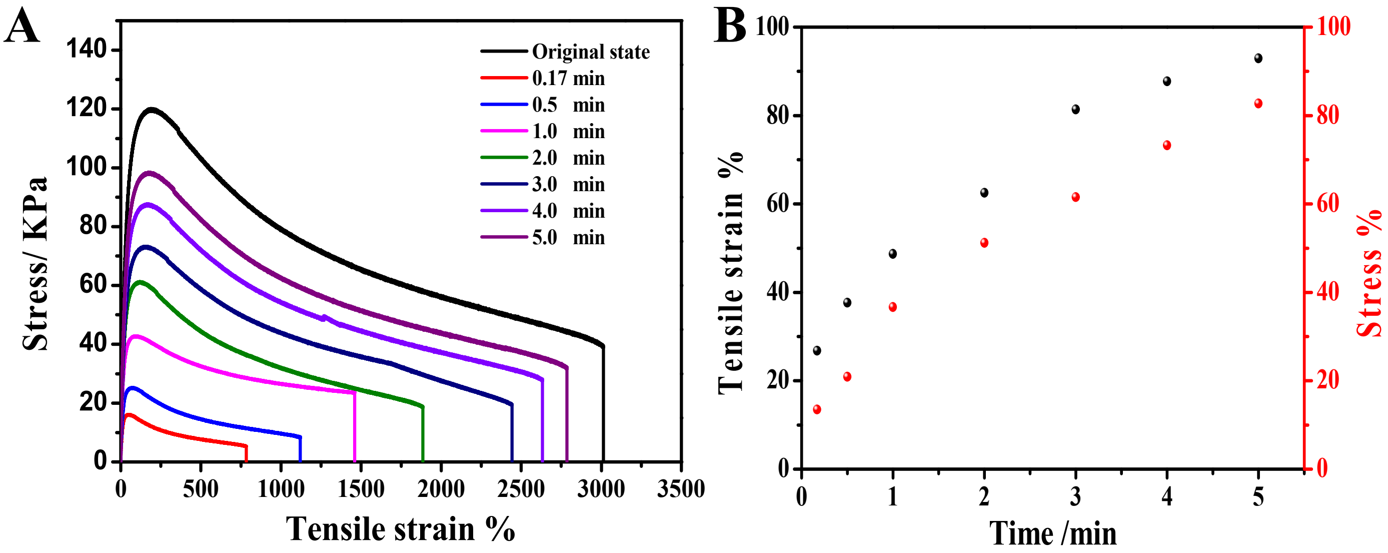

Figure S4. Tensile stress–strain curves of CMA-3 hydrogel during the self-healing process at different healing time (**A**,**B**).

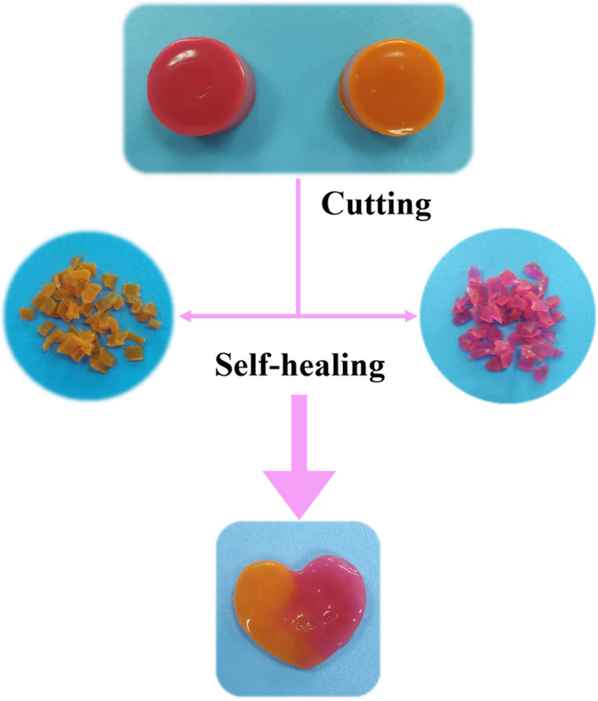

Figure S5. Photographs of self-healing ability of CMA-3 hydrogel.

Reference

1. Ge, G.; Lu, Y.; Qu, X.; Zhao, W.; Ren, Y.; Wang, W.; Wang, Q.; Huang, W.; Dong, X. Muscle-Inspired Self-Healing Hydrogels for Strain and Temperature Sensor. *ACS Nano* **2020**, *14*, 218–228.
2. Shin, S.-H.; Lee, W.; Kim, S.; Lee, M.; Koo, J.M.; Hwang, S.Y.; Oh, D.X.; Park, J. Ion-conductive self-healing hydrogels based on an interpenetrating polymer network for a multimodal sensor. *Chem. Eng. J.* **2019**, *371*, 452–460.
3. Chang, S.; Wang, B.; Liu, Y.; Li, Z.; Hu, X.; Zhang, X.; Zhang, H. Radiation-assistant preparation of highly conductive, transparent and self-healing hydrogels with triple-network structure. *Polymer* **2020**, *188*, 122156.
4. Ji, F.; Li, J.; Zhang, G.; Lan, W.; Sun, R.; Wong, C.P. Alkaline monomer for mechanical enhanced and self-healing hydrogels based on dynamic borate ester bonds. *Polymer* **2019**, *184*, 121882.
5. Ma, A.; Wang, G.; Yang, Z.; Bai, L.; Chen, H.; Wang, W.; Yang, H.; Wei, D.; Yang, L. Fabrication of Janus graphene oxide hybrid nanosheets by Pickering emulsion template for self-healing nanocomposite hydrogels. *Chem. Eng. J.* **2020**, *385*, 123962.
6. Bai, L.; Jiang, X.; Sun, Z.; Pei, Z.; Ma, A.; Wang, W.; Chen, H.; Yang, H.; Yang, L.; Wei, D. Self-healing nanocomposite hydrogels based on modified cellulose nanocrystals by surface-initiated photoinduced electron transfer ATRP. *Cellulose* **2019**, *26*, 5305–5319.
7. Pang, J.; Wang, L.; Xu, Y.; Wu, M.; Wang, M.; Liu, Y.; Yu, S.; Li, L. Skin-inspired cellulose conductive hydrogels with integrated self-healing, strain, and thermal sensitive performance. *Carbohydr. Polym.* **2020**, *240*, 116360.
8. Zheng, C.; Lu, K.; Lu, Y.; Zhu, S.; Yue, Y.; Xu, X.; Mei, C.; Xiao, H.; Wu, Q.; Han, J. A stretchable, self-healing conductive hydrogels based on nanocellulose supported graphene towards wearable monitoring of human motion. *Carbohydr. Polym.* **2020**, *250*, 116905.
9. Maryam, T.; Ali, P.; Maryam, A.; Hamid, T.; Seyyed, J.S.T.; Monireh, A.; Navid, R.; Mojtaba, B.; Rajender, S.V. An environmentally friendly wound dressing based on a self-healing, extensible and compressible antibacterial hydrogel. *Green Chem.* **2021**, *23*, 1312–1329.
10. He, X.L.; Sun, X.Q.; Meng, H.Y.; Deng, S.Y.; He, T.T.; Zang, H.J.; Wei, D.S., Self-healing polymeric ionic liquid hydrogels with high mechanical strength and ionic conductivity. *J. Mater. Sci.* **2021**, *56*, 1023.
11. Li, Y.Y.; Zhou, T.Y.; Yu, Z.Y.; Wang, F.; Shi, D.J.; Ni, Z.B.; Chen, M.Q., Effects of surfactant and ionic concentration on properties of dual physical crosslinking self-healing hydrogels by hydrophobic association and ionic interactions. *New J. Chem.* **2020**, *44*, 4061–4070.
12. Ye, L.; Lv, Q.; Sun, X.Y.; Liang, Y.Z.; Fang, P.W.; Yuan, X.Y.; Li, M.; Zhang, X.Z.; Shang, X.F.; Liang, H.Y., Fully physically cross-linked double network hydrogels with strong mechanical properties, good recovery and self-healing properties. *Soft Matter.* **2020**, *16*, 1840–1849.
